# Characterization of the complete plastid genome of *Astelia australiana* (J. H. Willis) L. B. Moore (Asteliaceae, Asparagales)

**DOI:** 10.1080/23802359.2019.1711233

**Published:** 2020-01-16

**Authors:** Michael D. Amor, Gareth D. Holmes, Elizabeth A. James

**Affiliations:** Royal Botanic Gardens Victoria, South Yarra, Australia

**Keywords:** *Astelia australiana*, rare species, conservation, plastid, disjunct

## Abstract

*Astelia australiana* is a robust understorey plant with a highly restricted distribution in southeastern Australia. Here we report its complete plastid genome. The genome was 157,943 bp in length and comprises a pair of inverted repeats (IRs) of 27,028 bp separated by a large single-copy region (LSC) of 85,699 bp, and a small single-copy region (SSC) of 18,188 bp. The GC content was 37.7%. In total, 132 genes were annotated including 81 protein-coding genes (PCGs), 38 tRNA genes, and 8 rRNA genes. Phylogenetic analysis of the PCGs from *A. australiana* aligned with those from 10 Asparagales representatives confirms that, based on these taxa, *A. australiana* is sister to *A. pumila* and sits within the Asteliaceae.

*Astelia* Banks & Sol. ex R.Br. is the largest genus within Asteliaceae with an Austral-Pacific distribution centered in New Zealand (Birch 2015). Three of the 26 species are endemic to eastern Australia (VicFlora 2016). Listed as vulnerable (EPBC Act 1999), *Astelia australiana* (J. H. Willis) L. B. Moore, is a tufted, rhizomatous perennial species confined to the temperate rainforest in two disjunct areas of southern Victoria (VicFlora 2016). As disjunction has been identified as one of the strongest predictors of genetic differentiation among Australian flora (Broadhurst et al. 2017), determining patterns of genetic variation in *A. australiana* will assist in identifying potentially distinct evolutionarily significant units (ESUs, Moritz 1994) and inform appropriate management strategies.

Here, we report and characterize the plastid genome sequence of *A. australiana* (GenBank accession MN839533) to provide a resource to promote the species’ conservation. This resource will enhance the assessment of infraspecific diversity, phylogeography and the distribution of maternal lineages.

Fresh leaf material was sampled from a cultivated plant originally sourced from Pioneer Creek, Victoria, Australia ( 37°53.95′S, 145°47.48′E, MEL

2475665). Genomic DNA was extracted from silica gel dried material using the CTAB protocol of an ISOLATE II Plant DNA Kit (Bioline Australia, Alexandria, Australia). Genome library preparation using a TruSeq Nano DNA Library Prep kit (Illumina, San Diego, CA) and 150 bp PE sequencing on an Illumina NovaSeq 6000 platform was undertaken at the Australian Genome Research Facility, Parkville.

We aligned the plastid genomes of three *Astelia pumila* individuals (MH752981.1, MH752984.1, MH752983.1) and generated a consensus sequence using Geneious 9.1.8 (https://www.geneious.com). We indexed this consensus sequence and mapped raw *A. australiana* reads to it using Bowtie-2 (Langmead et al. 2009). File conversion (from SAM to BAM, then from BAM to FASTQ) was performed using SAMtools (http://github.com/samtools/samtools). The read depth of mapped reads was normalized using BBNorm (sourceforge.net/projects/bbmap/) targeting 50 reads per site. Putative chloroplast reads were used for a *de novo* assembly in Geneious. The assembled *A. australiana* chloroplast contigs were aligned to the *A. pumila* reference genome and corrected manually when necessary. The close relationship and sequence similarity between *A. pumila* and *A. australiana* allowed us to transfer annotations directly from the reference genome.

The complete plastid genome of *A. australiana* was 157,942 bp long and comprised a pair of inverted repeats (IRs) of 27,028 bp separated by a large single-copy region (LSC) of 85,698 bp and a small single-copy region (SSC) of 18,188 bp. GC content was 37.7%. In total, 132 genes were annotated (81 PCGs, 38 tRNA genes, and 8 rRNA genes) including 114 unique genes and 18 duplicated genes in the IR. Synteny was maintained between the *A. australiana* genome, reported here, and the reference (*A. pumila*) with *ycf1* and *rpl22* recorded at the boundaries of the IRs.

The phylogenetic tree presented (Figure 1) illustrates *A. australiana* as grouped in the Asteliaceae with strong bootstrap support and sister to *A*. *pumila*, a species native to forest and wetland areas in Chile and Argentina.

**Figure 1.**
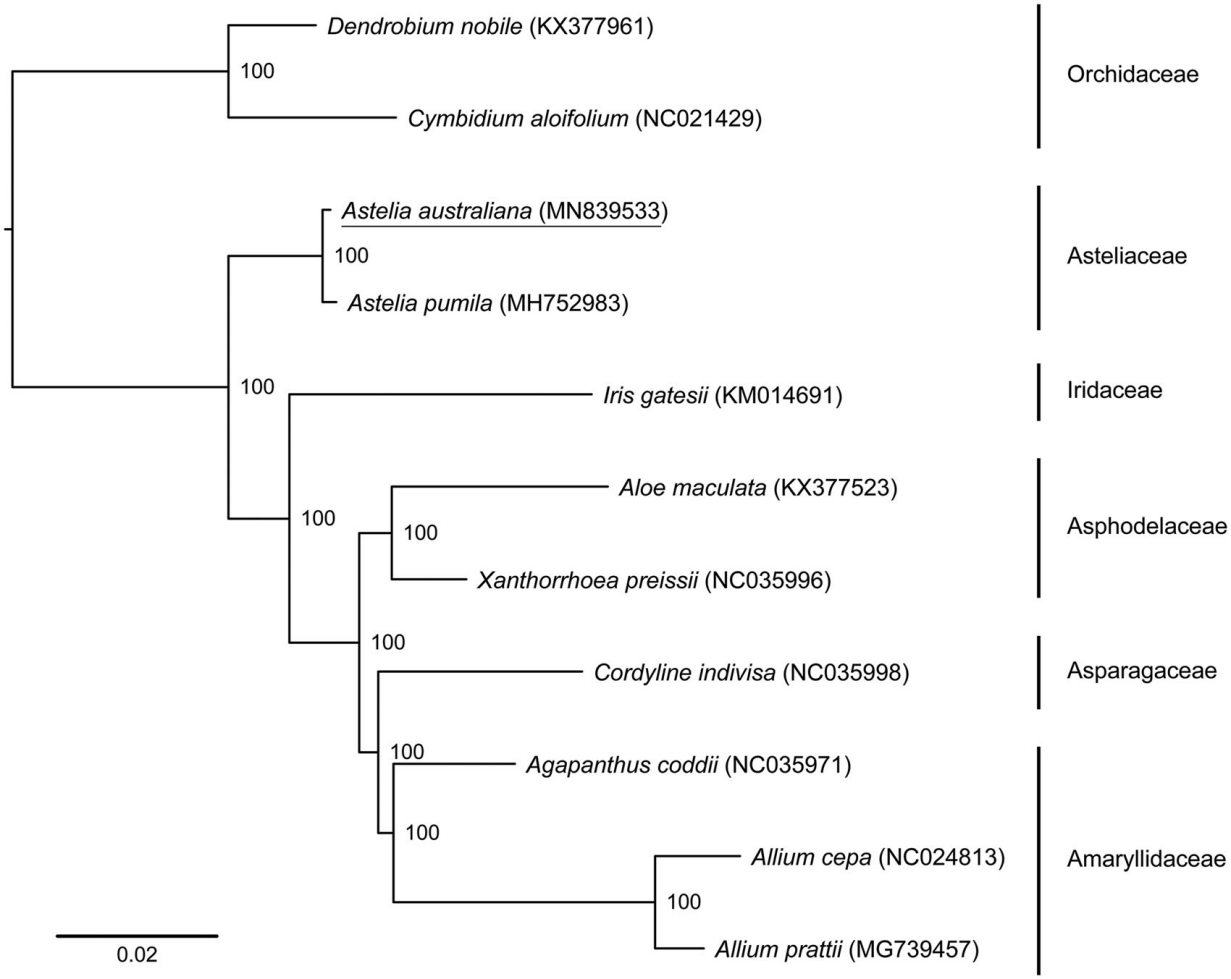
Maximum-likelihood topology depicting the relationships of *Astelia australiana* (family Asteliaceae) and several of its close relatives for which whole genome plastid data are published. The analysis was based on an alignment containing 69,870 bp of protein-coding sequence data representing 81 chloroplast genes from 11 accessions within the order Asparagales. The family Orchidaceae was used to root the phylogeny. Bootstrap support values are shown at nodes. Analysis was performed using the GTR + G model with 1000 rapid bootstrap iterations implemented in RAxML v8.2.12 (Stamatakis 2014). Gene deletions observed in accessions NC021429 (*petN*, *psbM*), KX377961 (*ndhK*, *ndhC*, *ndhI*), NC035998 (*clpP*) and KX377523 (*rpl32*) were replaced by N’s in the alignment file. The alignment used to produce this phylogeny is available via Mendeley data (http://dx.doi.org/10.17632/8rggb38ybx.1).
